# Conservative Management of a Symptomatic Anterior Limbus Vertebra in a Non-athletic Patient: A Case Report

**DOI:** 10.7759/cureus.112136

**Published:** 2026-07-06

**Authors:** Eric Chun-Pu Chu, Wing Ka Cheng, Eden YT Chu, Wai Ting Lee

**Affiliations:** 1 Chiropractic and Physiotherapy Centre, New York Medical Group, Hong Kong, CHN

**Keywords:** chiropractic manipulation, chiropractor, conservative management, disc herniation, intervertebral disc degeneration, limbus vertebra, low back pain, lumbar spondylosis, physiotherapy

## Abstract

Limbus vertebra (LV) is a frequently underdiagnosed spinal anomaly resulting from intraosseous herniation of nucleus pulposus material during skeletal immaturity, often discovered incidentally but capable of causing chronic low back pain (LBP) and neurological symptoms. We present the case of a sedentary adult with persistent LBP and paresthesia, whose imaging revealed an anterior LV at L5/S1 with concomitant intervertebral disc degeneration. Unlike most reported cases involving athletes, this case highlights LV as a cause of symptoms in non-athletic populations. The patient achieved significant relief through conservative management with chiropractic interventions, supporting a non-surgical approach as first-line therapy. A narrative review of current literature underscores the importance of accurate diagnosis-distinguishing LV from fractures, degenerative changes, and neoplastic or infectious processes-while emphasizing conservative measures for symptom control and functional recovery. Clinicians should consider LV in the differential diagnosis of chronic LBP, regardless of patient activity level, to guide effective management and avoid unnecessary interventions.

## Introduction

Limbus vertebra (LV) refers to a marginal intraosseous herniation of the nucleus pulposus, first described by Schmorl in 1927 as an intrabody disc herniation typically arising during childhood or adolescence [1]. Radiographically, LV is characterized by a well-circumscribed, triangular bony fragment with sclerotic margins, most commonly located at the anterosuperior corner of a lumbar vertebral body [2]. Although traditionally considered an incidental and clinically insignificant finding, LV has often been misdiagnosed as a vertebral fracture, infection, or tumor until pathological studies confirmed its discal origin [2]. Pathological and imaging studies have confirmed the discal origin of LV, with histological examinations of excised limbus fragments revealing the presence of nucleus pulposus material embedded within the osseous structure, often accompanied by cartilaginous tissue consistent with intrabody herniation [3,4]. Additionally, discography studies have provided direct evidence by demonstrating that contrast medium injected into the nucleus pulposus extends around the limbus fragment, verifying the herniation pathway [5]. The location of the LV is critical: anterior LV (ALV), the most common form, is implicated in accelerated intervertebral disc degeneration (IDD) and chronic low back pain (LBP) due to ongoing mechanical stress and instability at the disc-vertebra interface [6,7], whereas posterior LV (PLV) poses a higher risk of neurological symptoms, such as radiculopathy, from potential nerve root or spinal cord compression [8]. This distinction underscores the need for precise imaging and clinical correlation to differentiate LV from other pathologies.

The development of LV is linked to herniation of disc material during the period of vertebral endplate ossification (ages 6 to 20 years), with contributing factors including chronic mechanical stress, trauma, congenital abnormalities, and genetic predispositions such as COL11A1 polymorphisms [9,10]. Much of the existing literature has focused on athletic populations [6,7], yet LV in sedentary individuals-potentially exacerbated by poor posture and underlying IDD-remains underrecognized. Coexistence with Schmorl’s nodes or Scheuermann’s disease is also frequent, reflecting a shared vulnerability of the vertebral endplates [11,12]. While ALV has historically been dismissed as an incidental variant, growing biomechanical and clinical evidence demonstrates its active role in generating chronic LBP. The marginal separation of the bony fragment disrupts the structural integrity of the vertebral endplate, altering axial load distribution across the functional spinal unit. This mechanical instability accelerates IDD at the affected segment. The chronic mechanical strain, combined with micro-instability at the disc-vertebra interface, serves as a direct source of nociceptive LBP, making its recognition vital for primary-contact clinicians evaluating persistent spinal complaints.

This case report describes a symptomatic ALV in a sedentary adult successfully managed with chiropractic care and is accompanied by a narrative literature review on the pathophysiology, diagnosis, and management of LV. The aim is to highlight LV’s significance in the differential diagnosis of chronic LBP, especially in non-athletic populations, and to advocate for conservative, non-surgical approaches where appropriate.

## Case presentation

A 29-year-old female office worker presented with a longstanding history of LBP that had increased in severity over the past three months. She described the pain as a persistent, dull ache localized to her right lower back, rated at 7 out of 10 on the Visual Analog Scale (VAS), occasionally accompanied by pins-and-needles sensations radiating into her right anterior thigh and sometimes the leg. Her symptoms were aggravated by prolonged sitting-often 8 to 10 hours daily at her workstation-walking, and climbing stairs, and were partially relieved by rest and hot showers. This pain and associated paresthesia significantly limited her ability to perform work duties, engage in household chores, and participate in recreational activities. She reported frequent sleep disturbances due to discomfort and difficulty maintaining focus at work, contributing to irritability and decreased productivity.

On initial assessment, her quality of life was notably impaired. Previous conservative treatments-including non-steroidal anti-inflammatory drugs (NSAIDs), physiotherapy, and acupuncture-provided only temporary relief. She denied any history of trauma, systemic illness, or prior spinal surgery.

A comprehensive chiropractic physical examination was conducted. On inspection, she demonstrated a mild right-sided pelvic tilt and increased lumbar lordosis. Palpation revealed tenderness and mild muscle spasm over the L4, L5, and S1 spinous processes, as well as in the right paraspinal and gluteal musculature. Lumbar spine range of motion was within normal limits for flexion and lateral flexion, but extension elicited pain in the lower back. Orthopedic testing showed a negative straight-leg raise and sacral thrust, but the Valsalva maneuver provoked mild paresthesia in the right lower extremity. Neurological examination revealed intact motor strength and deep tendon reflexes in both lower limbs, with no sensory loss to light touch or pinprick. Gait assessment was normal, but the patient demonstrated guarded movement when transitioning from sitting to standing. Postural inspection demonstrated forward head posture, rounded shoulders, and a mild right-sided pelvic tilt. No standardized postural photography or quantitative angular measurements were obtained. No red flag findings were present. Radiological examination of the lumbar spine was then arranged to rule out major pathology.

**Figure 1:**
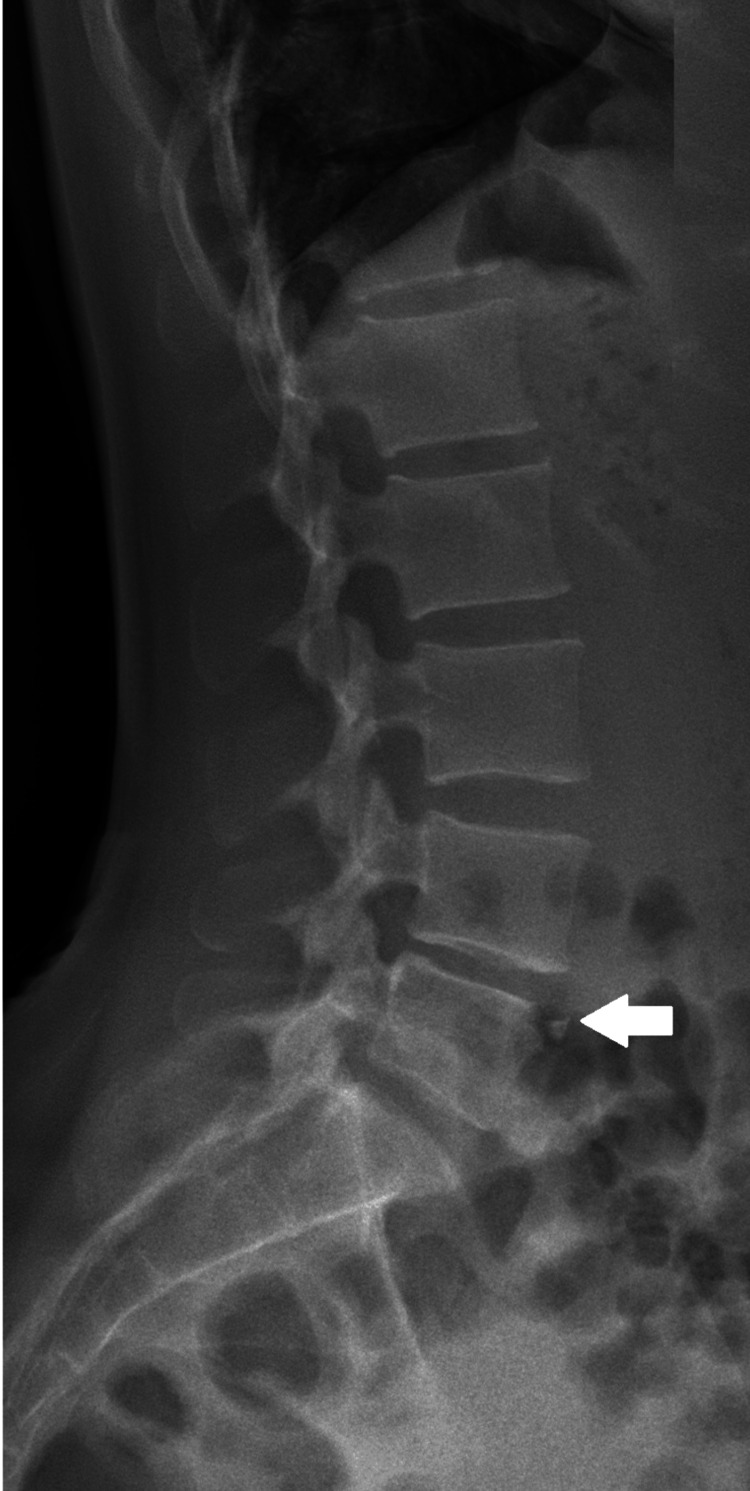
Sagittal plain radiograph of the lumbar spine demonstrating a limbus vertebra Standing lateral lumbar radiograph demonstrated an anterior limbus fragment at the anterosuperior corner of L5. L5/S1 disc height appeared preserved without definite narrowing (white arrow). There was possible mild posterior disc space narrowing at L4/L5 and minimal sclerosis at the posterior superior endplate of L5. No spondylolisthesis was observed.

Radiographic imaging of the lumbar spine (Figure 1) demonstrated reduced disc height at the L5/S1 level, consistent with IDD. Additionally, a triangular bony fragment with sclerotic margins was identified at the anterosuperior aspect of the L5 vertebral body, diagnostic of ALV. No magnetic resonance imaging (MRI) or computed tomography (CT) was pursued due to the absence of red flag symptoms (e.g., progressive weakness, bowel/bladder dysfunction, or history of trauma). The patient's sedentary occupation and associated postural stressors, combined with L5/S1 IDD, were hypothesized to exacerbate mechanical stress on the LV, contributing to her symptomatic presentation. Plain radiography objectively demonstrated reduced L5/S1 disc height and an anterosuperior limbus fragment; no additional postural metrics were collected.

Chiropractic treatment was initiated, consisting of instrument-assisted manipulation (using an Activator device) targeting the pelvic region to enhance flexibility and joint mobility, along with mechanical lumbar traction to decompress the L5/S1 disc space (28 kg force, L5-S1, 40:30 hold-to-rest ratio, 5° aslope) for 15 minutes per session. The Activator Method employs a handheld, spring-loaded instrument that delivers a low-force, high-acceleration mechanical impulse over a very short excursion without joint cavitation. Home exercises focused on core strengthening, including planks and pelvic tilts, to improve spinal stability. The treatment plan included three visits per week for the first month, followed by two visits per week for the next two months. Over three months of regular chiropractic care, the patient experienced substantial clinical and functional improvement. Her VAS pain score decreased from 7/10 to 2/10. She regained the ability to walk for over 30 minutes without pain or numbness, resumed stair climbing, and was able to complete full workdays without interruption or discomfort. She reengaged in light physical activities such as yoga and brisk walking, and her sleep quality improved, allowing for restful, uninterrupted nights. She reported enhanced energy, improved mood, and greater satisfaction with both work and quality of life. Monthly maintenance chiropractic care was scheduled to support continued progress and prevent symptom recurrence, and no adverse events occurred during her treatment. At the sixth month in-clinic review, she reported sustained improvement without recurrence requiring additional care.

## Discussion

Based on the patient’s sedentary work patterns and observed postural tendencies, we hypothesize that repetitive flexion/axial loading may have contributed to symptom generation at the L5/S1 level in the presence of an ALV. While LV is frequently reported in young athletes exposed to repetitive spinal stress [6,7], this case underscores its clinical relevance in non-athletic populations. The patient’s chronic symptoms, likely exacerbated by L5/S1 IDD and mechanical stress from prolonged sitting, align with the growing recognition of LV as a contributor to non-specific LBP across a broader demographic [2]. Importantly, the patient’s significant improvement following chiropractic intervention reinforces the role of conservative management as a first-line strategy for symptomatic LV.

Pathophysiology and etiology

LV results from intraosseous herniation of nucleus pulposus material between the ring apophysis and vertebral body during skeletal immaturity-a period when the apophyseal ring ossifies (ages 6-9) and fuses with the vertebral body by late adolescence (18-20 years) [13,14]. This process leads to the formation of a distinct, often triangular, osseous fragment. The etiology is multifactorial, involving biomechanical factors such as repetitive flexion loading, genetic predispositions (e.g., the COL11A1 TT genotype decreasing growth plate resistance) [9], developmental anomalies, and environmental influences including both high-impact sports and sedentary lifestyle [15,16]. The anterosuperior vertebral margin is the most frequent site, likely due to anatomical and mechanical factors [2]. LV shares a pathophysiological basis with Schmorl’s nodes and Scheuermann’s disease, with up to one-third of cases exhibiting overlapping features [11,12]. In this case, prolonged sedentary posture may have replicated the chronic mechanical stress typically seen in athletes, contributing to both IDD and symptomatic LV [17].

Clinical presentation and diagnosis

Although LV is often asymptomatic and incidentally discovered [18], symptomatic presentations may include non-specific LBP, muscle spasm, spinal stiffness, or-particularly in PLV-radiculopathy [8,19]. Such symptoms can mimic other spinal diseases, including vertebral fractures, inflammatory spondyloarthropathies, infections, and neoplasms, necessitating accurate and thorough diagnostic evaluation [14]. In pediatric and adolescent populations, irregular or fragmented appearances may lead to confusion with more aggressive pathologies [2].

Imaging plays a central role in diagnosis. Radiographs are typically the first-line modality and may reveal a well-circumscribed, sclerotic, triangular fragment adjacent to a vertebral body defect [2]. CT imaging further delineates fragment morphology and is particularly valuable for identifying PLV [8]. MRI is essential for assessing associated disc pathology, ruling out acute fractures (via absence of bone marrow edema), and identifying concurrent lesions such as Schmorl’s nodes [2]. The differential diagnosis is broad and includes fractures (which lack sclerotic margins), osteophytes (seen in degenerative contexts), disc or ligamentous calcifications, infections (associated with contrast enhancement), and tumors such as meningiomas (with characteristic imaging features) [19]. In this patient, conventional radiography was sufficient, and the imaging change has been associated with LBP, likely reflecting a local degenerative and inflammatory process that sensitizes lumbar nociceptors in the affected spinal segment.

Management and outcomes

Conservative management is generally the cornerstone of treatment for symptomatic LV, focusing on pain relief, restoration of function, and prevention of recurrence [2]. The 2023 WHO guideline recommends structured education, exercise therapies, manual and psychological therapies, and NSAIDs, while discouraging routine use of opioids, muscle relaxants, injectable anesthetics, and many passive or pharmacological treatments [20,21]. Chiropractic techniques, such as spinal manipulation and traction, can address mechanical dysfunction and provide symptomatic relief, as illustrated by this patient’s reduction in pain from a VAS score of 7/10 to 2/10 following treatment [22,23]. Outcomes tend to be favorable with conservative measures, especially in cases without large apophyseal fragments or significant neurological compromise; larger fragments may be associated with less optimal results [2].

Surgical intervention is reserved for refractory cases, particularly those involving PLV with persistent nerve root compression. Surgical options include laminectomy, discectomy, targeted fragment removal, spinal fusion, vertebroplasty, kyphoplasty, and, in select cases, minimally invasive or robotic-assisted approaches for enhanced precision [24]. The use of advanced preoperative planning tools, such as 3D-printed models and artificial intelligence (AI)-assisted software, can help mitigate operative risks in complex cases [25]. Literature consistently reports favorable outcomes, with conservative approaches being successful in 80%-90% of patients without compressive symptoms [26].

Limitations

This case report is limited by its single-patient design and the lack of long-term follow-up. We did not collect standardized, quantitative postural measures (e.g., photogrammetry for craniovertebral angle, Cobb L1-S1 lordosis, or sacral slope), nor did we include posture photographs; therefore, posture-related interpretations should be considered exploratory. In addition, we did not obtain an MRI or perform interventional diagnostic procedures; therefore, we cannot determine the specific contribution of the limbus fragment versus disc degeneration or other structures. The observed clinical improvement with conservative care is non-specific and should not be interpreted as evidence of causation. Nonetheless, it adds to the growing body of evidence supporting the efficacy of chiropractic and conservative therapies for LV-associated LBP, particularly in non-athletic populations.

Given the limitations inherent in single-case designs and the diagnostic challenges surrounding symptomatic vs. asymptomatic structural variants, future large-scale prospective cohort studies are needed to establish the true prevalence of symptomatic ALV in non-athletic, sedentary populations. Longitudinal research utilizing advanced cross-sectional modalities, such as quantitative CT or functional MRI, would provide valuable insight into the exact rate of accelerated IDD adjacent to the limbus fragment over time. Furthermore, randomized controlled clinical trials comparing multi-modal manual therapy, structured core stabilization protocols, and standard medical management are warranted to identify optimal, standardized conservative care pathways and evaluate long-term functional outcomes for these patients.

## Conclusions

This case demonstrates that LV can be a significant contributor to chronic LBP and paresthesia in sedentary adults, particularly when associated with IDD, and that targeted conservative management-including chiropractic care-can yield substantial clinical improvement. The accompanying literature review reinforces the importance of recognizing LV as a diagnostic consideration in patients with persistent LBP, regardless of athletic status, to prevent misdiagnosis and unnecessary interventions. Future research should focus on elucidating genetic risk factors and comparing the efficacy of various conservative and surgical therapies across diverse patient populations.

## References

[1] Shim MR (2019). Limbus vertebra and low back pain: a case report and review of literature. Int J Sports Exerc Med.

[2] Nișcoveanu C, Refi D, Obada B, Dragosloveanu S, Scheau C, Baz RO (2024). Beyond the bony fragment: a review of limbus vertebra. Cureus.

[3] Laredo JD, Bard M, Chretien J, Kahn MF (1986). Lumbar posterior marginal intra-osseous cartilaginous node. Skeletal Radiol.

[4] Wang YB, Chen SL, Cao C, Zhang K, Liu LM, Gao YZ (2020). Percutaneous transforaminal endoscopic discectomy and fenestration discectomy to treat posterior ring apophyseal fractures: a retrospective cohort study. Orthop Surg.

[5] Ghelman B, Freiberger RH (1976). The limbus vertebra: an anterior disc herniation demonstrated by discography. AJR Am J Roentgenol.

[6] Koyama K, Nakazato K, Min SK, Gushiken K, Hatakeda Y, Seo K, Hiranuma K (2013). Anterior limbus vertebra and intervertebral disk degeneration in Japanese collegiate gymnasts. Orthop J Sports Med.

[7] Acosta V, Pariente E, Lara M, Pini SF, Rueda-Gotor J (2016). Limbus vertebra and chronic low back pain. J Fam Med.

[8] Huang PY, Yeh LR, Tzeng WS, Tsai MY, Shih TT, Pan HB, Chen CK (2012). Imaging features of posterior limbus vertebrae. Clin Imaging.

[9] Samanta A, Lufkin T, Kraus P (2023). Intervertebral disc degeneration-current therapeutic options and challenges. Front Public Health.

[10] Zhang J, Zhang W, Yue W, Qin W, Li Z, Xu G (2025). Adolescent lumbar disc herniation: etiology, diagnosis, and treatment options. J Orthop Surg Res.

[11] Chan PK, Shah K, Sahu A, Hogarth M (2025). Schmorl's nodes and vertebral fractures: a diagnostic dilemma. Radiol Case Rep.

[12] Wáng YX (2024). Cartilaginous endplate coverage of developmental Schmorl's node and the relevance of this in Schmorl's node etiology-based classification. Quant Imaging Med Surg.

[13] Akhaddar A, Belfquih H, Oukabli M, Boucetta M (2011). Posterior ring apophysis separation combined with lumbar disc herniation in adults: a 10-year experience in the surgical management of 87 cases. J Neurosurg Spine.

[14] Chu EC, Cheong BK, Chu EY (2025). Lumbar apophyseal ring fracture with central disc herniation: a case report. Cureus.

[15] Lin AF, Piong SZ, Wan WM, Li P, Chu VK, Chu EC (2023). Unlocking athletic potential: the integration of chiropractic care into the sports industry and its impact on the performance and health of athletes and economic growth in China and Hong Kong. Cureus.

[16] Chu EC, Lin AF, Mok S, Piong SZ, Ng G (2023). A central slip injury in a professional basketball player. Cureus.

[17] Sanal HT, Yilmaz S, Simsek I (2012). Limbus vertebra. Arthritis Rheum.

[18] Graikos G, Gkoudina A, Tsakonas N, Christakis N (2020). Limbus vertebrae as incidental finding in a patient with acute lower back pain. Cureus.

[19] Tuna S, Özdemir T, Öz HE (2016). Limbus vertebra presenting with inflammatory low back pain: a case report. J Clin Diagn Res.

[20] World Health Organization (2023). WHO Guideline for Non-surgical Management of Chronic Primary Low Back Pain in Adults in Primary and Community Care Settings. https://www.who.int/publications/i/item/9789240081789.

[21] Chu EC, Trager RJ (2022). Effectiveness of multimodal chiropractic care featuring spinal manipulation for persistent spinal pain syndrome following lumbar spine surgery: retrospective chart review of 31 adults in Hong Kong. Med Sci Monit.

[22] Ng GS, Lee LY, Chu EC (2023). Undiagnosed osteoporotic vertebral fractures in an octogenarian during the coronavirus disease pandemic. Cureus.

[23] Chan AK, Ng GS, Cheong BK, Ng KK, Chu EC (2023). Sacral chordoma presenting as back pain in the chiropractic clinic: a case report. Cureus.

[24] Pérez de la Torre RA, Ramanathan S, Williams AL, Perez-Cruet MJ (2022). Minimally-invasive assisted robotic spine surgery (MARSS). Front Surg.

[25] Öztürk AM, Süer O, Govsa F, Özer MA, Akçalı Ö (2022). Patient-specific three-dimensional printing spine model for surgical planning in AO spine type-C fracture posterior long-segment fixation. Acta Orthop Traumatol Turc.

[26] Chang CH, Lee ZL, Chen WJ, Tan CF, Chen LH (2008). Clinical significance of ring apophysis fracture in adolescent lumbar disc herniation. Spine (Phila Pa 1976).

